# Sex Modulates Cardiovascular Effects of Icodextrin-Based Peritoneal Dialysis Solutions

**DOI:** 10.3389/fphys.2022.911072

**Published:** 2022-05-23

**Authors:** Ramón Paniagua, Elvia García-López, Marcela Ávila-Díaz, María-de-Jesús Ventura, Oscar Orihuela, María-del-Carmen Prado-Uribe, Juan-Manuel Gallardo-Montoya, Bengt Lindholm

**Affiliations:** ^1^ Centro Médico Nacional Siglo XXI, Unidad de Investigación Médica en Enfermedades Nefrológicas, Hospital de Especialidades, Instituto Mexicano del Seguro Social, México, Mexico; ^2^ Karolinska Institut, Stockholm, Sweden; ^3^ Centro Médico Nacional Siglo XXI, Departamento de Cardiología, Hospital de Especialidades, Instituto Mexicano del Seguro Social, México, Mexico

**Keywords:** peritoneal dialysis, icodextrin, diabetes, α-amylase, blood volume, sex

## Abstract

**Background/Aims:** Some previous observations have noted that after six months of peritoneal dialysis (PD) treatment with icodextrin solutions, blood pressure (BP) and NT-proBNP tend to return to baseline values. This may be due to accumulation of icodextrin products that exert a colloid osmotic effect, which drives water into the bloodstream, causing the rise in blood pressure. Since icodextrin is metabolized by α-Amylase and its gene copies are lower in females than in males, we hypothesized icodextrin metabolites reach higher concentrations in females and that cardiovascular effects of icodextrin are influenced by sex.

**Methods:** Secondary analysis of a RCT comparing factors influencing fluid balance control in diabetic PD patients with high or high average peritoneal transport receiving icodextrin (*n* = 30) or glucose (*n* = 29) PD solutions. Serum icodextrin metabolites, osmolality, body composition and Inferior Vena Cava (IVC) diameter were measured at baseline, and at 6 and 12 months of follow-up.

**Results:** After six months of treatment, icodextrin metabolites showed higher levels in females than in males, particularly G5-7 and >G7, serum osmolality was lower in females. In spite of reduction in total and extracellular body water, ultrafiltration (UF) was lower and IVC diameter and BP increased in females, suggesting increment of blood volume.

**Conclusion:** Females undergoing PD present with higher levels of icodextrin metabolites in serum that may exert an increased colloid-osmotic pressure followed by less UF volumes and increment in blood volume and blood pressure. Whether this could be due to the lesser number of α-Amylase gene copies described in diabetic females deserves further investigation.

## Introduction

Icodextrin is a specific fraction of dextrin (a starch-derived water-soluble glucose polymer) that has been successfully used as a d-glucose substitute as colloid osmotic agent in peritoneal dialysis (PD) solutions. It does not easily diffuse through the peritoneal membrane which allows longer permanence in the peritoneal cavity exerting a sustained and more efficient peritoneal ultrafiltration (UF) (Ho-Dac-Pannekeet, et al., 1996; Krediet and Mujais, 2002). Since 1990s, icodextrin solution has been used to increase UF for a better management of patients with fluid overload reducing total body water (TBW), extracellular water (ECW) and improving blood pressure (Mistry, et al., 1994; Davies, et al., 2003). All these benefits have been consistently reproduced without compromising residual renal function or urine output (Htay, et al., 2018). Icodextrin solutions have additional advantages; they reduce peritoneal glucose exposure and absorption which in turn prolongs patient and PD technique survival (Han, et al., 2012), and facilitates metabolic control in diabetic patients (Szeto and Johnson, 2017; Wang, et al., 2015). These combined effects are particularly useful in diabetic patients with high/fast peritoneal solute transport rate among whom icodextrin increases UF without metabolic risk (Cueto-Manzano and Correa-Rotter, 2000).

In spite of its notably advantages, some less favorable changes were noted in patients treated with icodextrin solutions. Over six months of continuous use, patients show trend of blood pressure back to baseline levels, as well as increments in the circulating levels of atrial and brain natriuretic peptides, markers of expansion of blood volume and myocardial damage (Davies, et al., 2008; Bouchi, et al., 2006). Furthermore, in a randomized controlled trial comparing glucose PD solutions against icodextrin solutions, focusing on the glucose sparing effects of icodextrin, a higher rate of cardiovascular adverse events was noted (Li, et al., 2013). In some papers, authors have suggested accumulation of icodextrin metabolites as a plausible cause of this phenomenon; however, this has not been addressed (Bouchi, et al., 2006).

Icodextrin leaves peritoneal cavity and reaches blood stream via the lymphatic circulation, this account from 20 to 40% for the longer dwell of 8–16 h. In the circulation, icodextrin is hydrolyzed by circulating α-Amylase, yielding oligosaccharides (also called icodextrin metabolites) such as maltose (G2), maltotriose (G3), maltotetraose (G4) and maltopentaose (G5) and these molecules may accumulate over the long term (Davies, 1994; García-Lopez, et al., 2005; Garcia-Lopez et al., 2008). Based on this known mechanism, it can be assumed that production and accumulation of icodextrin metabolites in blood with potential capacity to exert colloid osmotic effect and increase blood volume depends of α-Amylase activity. Interestingly, the number of serum amylase gene copies has recently found associated with the risk of develop obesity and diabetes. The number of this gene and enzyme activity are reduced in diabetics and even more reduced in females (Viljakainen, et al., 2015; Nakajima, 2016; Al-Akl et al., 2021).

In this study, we address the hypothesis that icodextrin metabolites reach higher concentrations in female than in male diabetic patients undergoing Continuous Ambulatory Peritoneal Dialysis (CAPD) and those cardiovascular effects of icodextrin are influenced by sex.

## Materials and Methods

### Design

A secondary analysis was performed with data from a prospective, multicenter, randomized controlled trial of diabetic patients undergoing CAPD with high and high average peritoneal transport type in whom icodextrin-based solutions were compared with glucose-based solutions as regards ability to control hypertension and edema as signs of fluid overload and metabolic control (Paniagua, et al., 2009). Briefly, after randomization, all patients received three 2-L exchanges of 1.5% dextrose (Dianeal, Baxter) for the daytime dwells. For the long dwell, patients in the control group (*n* = 29) received at least one bag of 2.5% dextrose (Dianeal, Baxter) and patients in the icodextrin group (*n* = 30) received 7.5% icodextrin (Extraneal, Baxter). Liberal use of 2.5% or 4.25% dextrose solutions was allowed in both groups in order to reach the treatment goal. The study protocol was approved by the Local Research Committees of all the participating hospitals, and the National Commission for Scientific Research of the Instituto Mexicano del Seguro Social (IMSS).

### Patient Population

Patients were included if they had end stage renal disease secondary to diabetic nephropathy, if they had high or high average peritoneal transport type, and signed a written informed consent. Patients were excluded when seropositive for hepatitis B or HIV, if they had malignancies, or were receiving immunosuppressive medication. Patients who had had a peritonitis episode 1 month or less before being screened were also excluded. There was no selection by age, gender, residual renal function or time on dialysis. Patients were followed-up for 12 months (Paniagua, et al., 2009).

### Data Collection

Clinical and demographic data were collected at baseline from medical records. In-office blood pressure was measured every month; 24-h ambulatory blood pressure; body composition by electrical bioimpedance (Biodynamics, Seattle, WA, USA) and echocardiogram with Doppler (Toshiba Sonolayer ultrasound system. Toshiba Corporation, Tochigi-Ken, Japan) were taken at baseline, at 6 and 12 months, and inferior vena cava diameter calculated (Vicente-Martinez et al., 2004). Biochemical parameters including serum α-Amylase activity, as well as inflammation and cardiovascular biochemical variables were also recorded in the same periods. For the secondary analysis, additional biochemical measurements were made, among them icodextrin metabolites and osmolality of serum.

Plasma α-Amylase activity was measured using an adapted routine method using p -nitrophenol maltoheptaoside as a substrate (Anderstam, et al., 2003). Plasma icodextrin metabolites, G2-G7 were measured using gel filtration high-performance liquid chromatography as described elsewhere (García-Lopez, et al., 2005). The concentrations of G8 to G10 (if present) were estimated as they eluted successively at predictable elution times since no commercial standards are available (Garcia-Lopez et al., 2008). Serum osmolality was assessed by osmometer (Wescor 5520, USA), and serum sodium by ion selective electrode (ILyte Na^+^K^+^Cl^−^ system, Lexinton, MS, USA).

### Statistics

Data are expressed as percentages or mean ± SD according characteristics of variables. For statistical analysis, patients of each group were divided by gender and differences were analyzed by repeated comparisons over time. Pearson´s correlations were done between biochemical indices and cardiovascular variables. All statistical analysis was done with SPSSw v24.

## Results

A complete description of data at baseline is in the original paper (Paniagua, et al., 2009). Table 1 shows a summary of that data. There were no significant differences between glucose and icodextrin groups at baseline. Patients had a much-reduced residual renal function, but values of total and peritoneal Kt/V as well as peritoneal ultrafiltration were considered appropriate for dialysis adequacy.

**TABLE 1:** Demography, clinical and biochemical characteristics of patients at baseline.

Parameter	Glucose	Icodextrin	*p* Value
N	29	30	
Age (years)	60.5 ± 9.3	58.9 ± 7.9	0.47
Gender (n) (female/male)	13f/16 m	18f/12 m	0.30
Weight (kg)	61.5 ± 12.3	62.1 ± 10.4	0.85
Height (cm)	159.0 ± 13.7	156.6 ± 7.4	0.41
BMI (kg/m2)	24.10 ± 3.41	25.18 ± 3.74	0.70
Systolic BP (mmHg)	139.8 ± 29.4	148.9 ± 24.1	0.20
Diastolic BP (mmHg)	80.2 ± 16.4	84.9 ± 15.0	0.27
Total body water (% body weight)	59.49 ± 7.77	59.42 ± 7.44	0.90
Extracellular body fluid volume (% body weight)	26.95 ± 2.99	26.86 ± 3.02	0.70
Comorbidity			
Acute myocardial infarct	2	4	0.31
Stroke	2	2	0.68
Allergy	2	6	0.42
Serum albumin (g/dl)	2.71 ± 0.40	2.67 ± 0.48	0.70
Hemoglobin (g/dl)	10.6 ± 2.4	10.8 ± 2.2	0.71
Hematocrit (%)	31.6 ± 7.0	30.9 ± 6.5	0.72
Insulin use (n)	17	17	0.81
Serum glucose (mg/dl)	172 ± 152	164 ± 88	0.81
HbA1c (%)	7.59 ± 2.80	8.01 ± 2.80	0.57
Serum Sodium (mEq/L)	132.8 ± 3.68	133.04 ± 3.71	0.78
Time on PD (months)	19.6 ± 16.6	17.6 ± 14.1	0.61
D/P Cr PET 4 h	0.79 ± 0.09	0.81 ± 0.08	0.74
pCrCl (L/week)	54.1 ± 10.3	54.1 ± 11.2	0.99
rCrCl (L/week)^a^	0.90 | 2.73	0.70 | 5.70	0.14
Total CrCl (L/week)	55.73 ± 10.35	57.30 ± 9.94	0.63
pKt/V 6	1.90 ± 0.3	1.80 ± 0.40	0.22
rKt/V (L/week)^a^	0.12 | 0.40	0.14 | 0.08	0.17
Total Kt/V	1.92 ± 0.35	1.82 ± 0.38	0.30
r(CrCl + ureaCl)/2 (ml/minute)^a^	0.63 | 0.20	0.61 | 0.37	0.14
Urine volume (mL/24 h)	149 ± 255	253 ± 300	0.16
Peritoneal ultrafiltration (mL/24 h)	1414 ± 703	1271 ± 745	0.45

BMI, body mass index; BP , blood pressure, HbA1c = Glycated hemoglobin

a = median|IQR.

**FIGURE 1:**
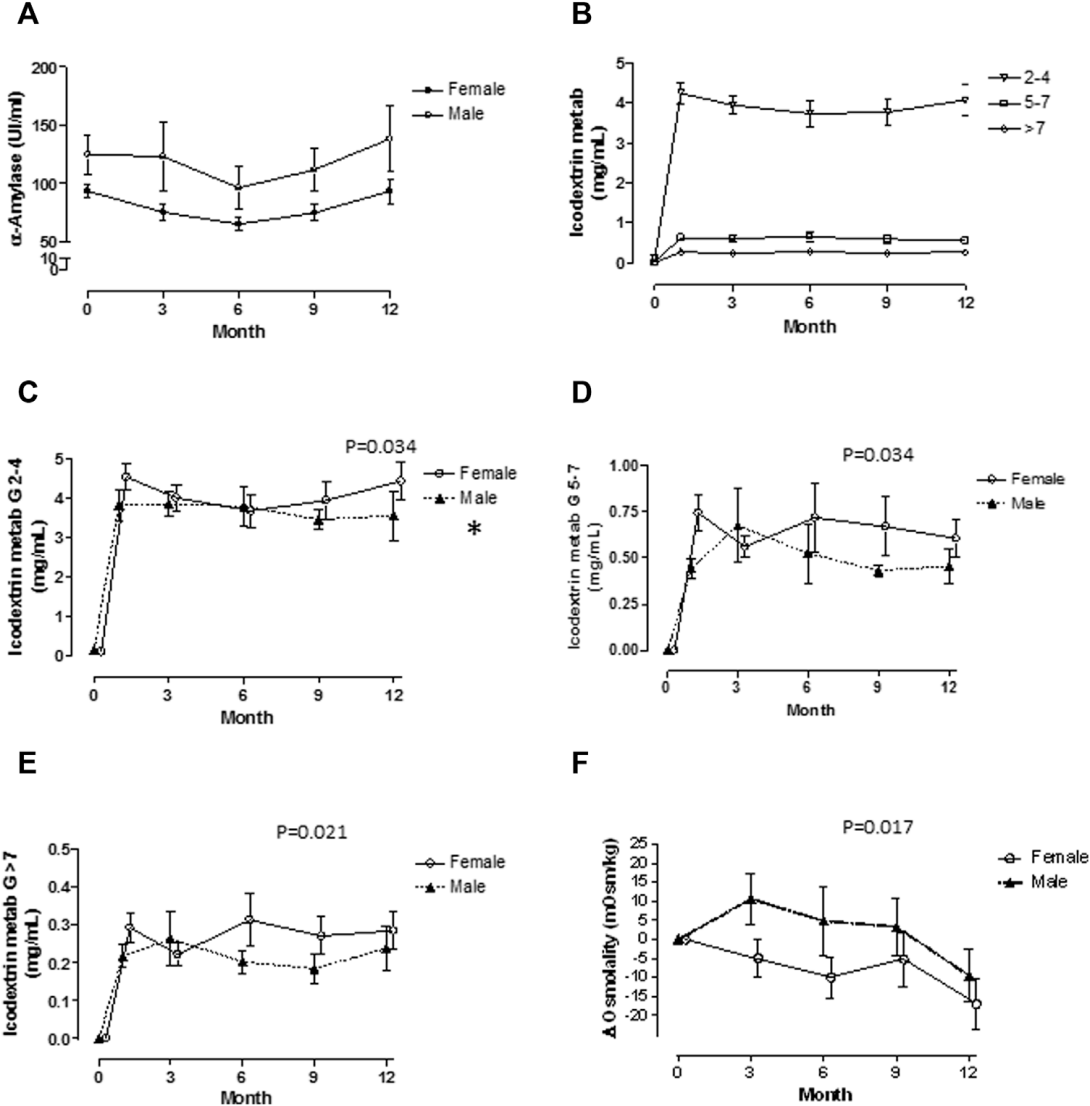
Biochemical variables are shown by sex. α-Amylase activity **(A)** was significantly lower in females. Plasma levels of Icodextrin metabolites were inversely related with its molecular size **(B)**. Plasma levels of G2-G4 were not different by sex **(C)**, but significant increments in G5-G7 **(D)** and >G7 **(E)**, were found in females over males after 6 months of treatment. Plasma osmolality was lower in females **(F)**.

Table 2 shows body fluid compartments, biochemical, renal, and cardiovascular data for the 30 patients in the icodextrin group at baseline by sex, 18 females and 12 males. As expected, body size parameters and body fluid composition were different between sexes, and icodextrin dose by BSA 10% higher in females. In both sexes, residual renal function was very low. Figure 1 shows selected biochemical variables. In panel A are values of serum α-Amylase activity showing that females had lower values in comparison with males. Panel B shows changes in icodextrin metabolites observed in the total number of patients: the highest concentrations were reached in the 2-4 glucose molecules fragment and the lowest in >7 molecules fragment. Considered as a whole group, the concentration of icodextrin fragments remained constant. Panels C to E show concentrations of different fragments divided by sex. Significantly higher concentrations in fragments G5-G7 and >G7 were noted in females after six months of treatment. Panel F shows changes in serum osmolality where female patients had lower values, which is opposite to increments in icodextrin fragments. Table 3 contains data of changes of serum glucose, urea, and serum sodium concentrations, as well as, osmolality, and total icodextrin fragments from baseline to 12 months. The average throughout the follow-up of total icodextrin fragments concentration is also shown. Urea increased in men and decreased in women while sodium decreased more significantly in women.

**TABLE 2:** Cardiovascular vaiables by sex at baseline.

Sex	Female	Male	Total	p
n	18	12	30	—
Age (year)	59.39 ± 8.66	58.08 ± 6.88	58.87 ± 7.90	0.665
Weight (kg)	59.72 ± 11.27	65.69 ± 8.00	62.11 ± 10.37	0.124
Height (cm)	152.19 ± 5.12	163.17 ± 5.13	156.58 ± 7.43	0.000
Body mass index (kg/m^2^)	25.61 ± 4.42	24.54 ± 2.44	25.18 ± 3.74	0.405
Body surface area (m^2^)	1.556 ± 0.146	1.708 ± 0.117	1.617 ± 0.153	0.010
Systolic blood pressure (mmHg)	153.56 ± 34.32	157.50 ± 23.01	155.13 ± 29.92	0.730
Diastolic blood pressure (mmHg)	85.50 ± 15.81	91.50 ± 14.32	87.90 ± 15.27	0.300
Serum glucose (mg/dl)	157.45 ± 73.31	179.32 ± 161.62	167.83 ± 122.72	0.515
Serum urea (mg/dl)	105.77 ± 26.73	119.54 ± 32.86	112.31 ± 30.34	0.082
Serum creatinine (mg/dl)	7.93 ± 2.16	10.45 ± 3.04	9.12 ± 2.89	0.005
Serum sodium (mEq/L)	134.47 ± 2.84	135.02 ± 2.94	134.70 ± 2.85	0.620
Serum osmolality (mOsm/kg)	296.59 ± 16.24	294.92 ± 27.66	295.90 ± 21.26	0.839
Protein intake (g/kg/day)	1.24 ± 0.37	1.14 ± 0.30	1.20 ± 0.35	0.450
r(CrCl + ureaCl)/2 (mL/min/1.73 m^2^)^a^	0.16 ± 0.26	0.41 ± 0.49	0.30 ± 0.40	0.056
Total body water (% of BW)	57.05 ± 7.89	62.98 ± 5.19	59.42 ± 7.44	0.019
Extracellular water (% of BW)	26.60 ± 3.45	27.26 ± 2.31	26.86 ± 3.02	0.568
Peritoneal ultrafiltration (mL/24 h)	1173.89 ± 485.23	1417.00 ± 1029.27	1271.13 ± 744.67	0.390
Left ventricle mass index (g/m^2^)	265.83 ± 92.89	298.46 ± 64.13	278.89 ± 82.96	0.299
Inferior Vena Cava diameter (mm)	12.14 ± 4.57	16.56 ± 5.29	14.48 ± 5.32	0.087
NT-proBNP (ng/ml)	4.87 ± 1.68	4.73 ± 2.40	4.82 ± 1.96	0.849
Icodextrin dose (g/m^2^)	97.25 ± 9.58	88.20 — 6.10	93.63 — 9.40	0.010

a = Residual renal function.

**TABLE 3:** Changes from baseline values of selected biochemical variables.

Variable	Change	Female	Male	p
Serum Glucose (mg/dl)	Δ 0 to 12 Months	−22.96 ± 21.78	−3.43 ± 40.58	0.674
BUN (mg/dl)	Δ 0 to 12 Months	−6.27 ± 6.89	24.81 ± 6.57	0.002
Serum Albumin (g/dl)	Δ 0 to 12 Months	−0.22 ± 0.17	−0.06 ± 0.13	0.442
Serum Sodium (mEq/L)	Δ 0 to 6 Months	−4.192 ± 1.08	−1.08 ± 0.826	0.035
Serum Osmolality (mOsm/L)	Δ 0 to 12 Months	−6.00 ± 2.73	−2.39 ± 1.83	0.287
ICO fragments (Total)	Δ 0 to 12 Months	5.343 ± 0.58	4.681 ± 0.41	0.366

**FIGURE 2:**
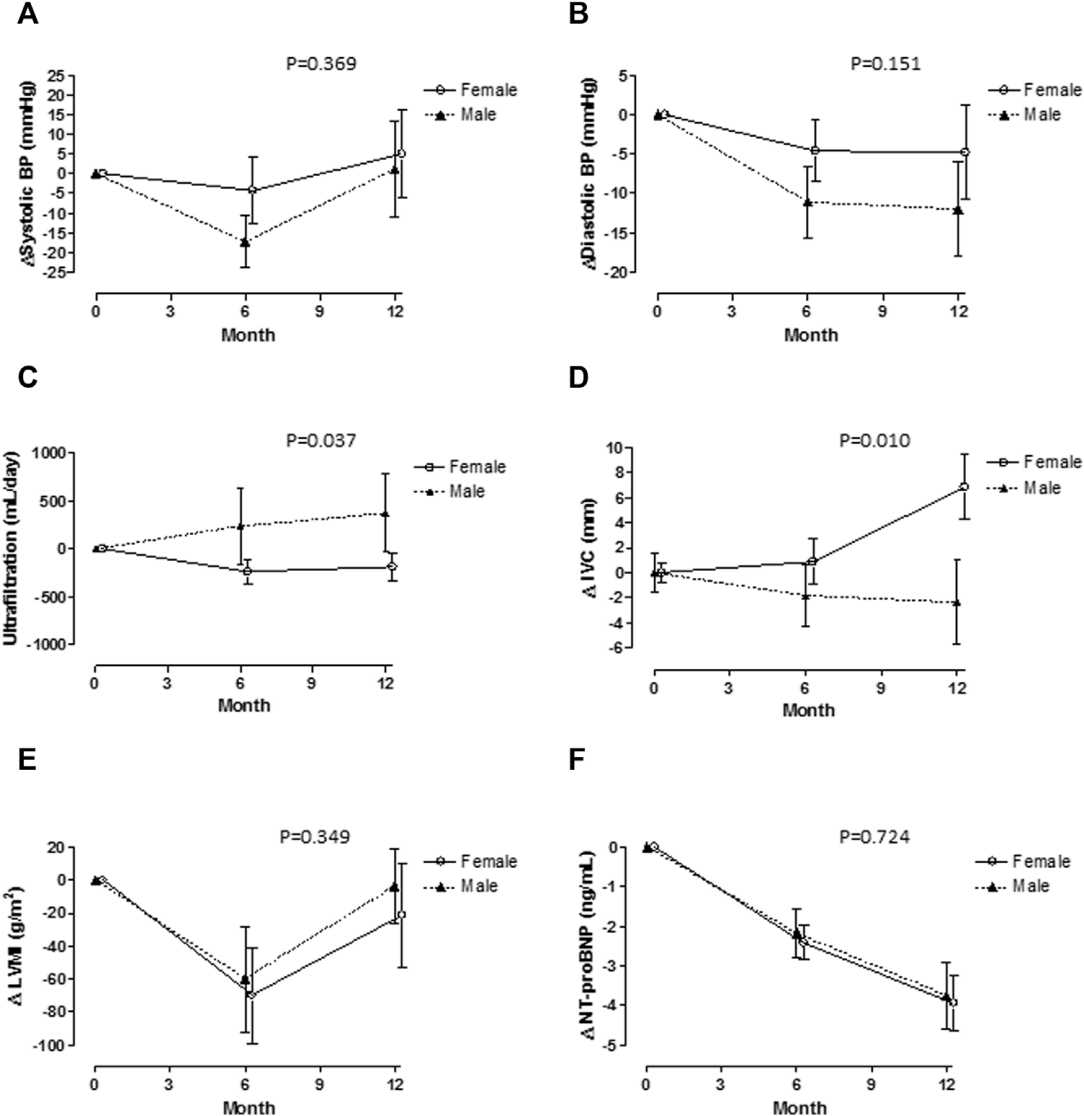
Cardiovascular variables are shown by sex. Systolic blood pressure **(A)** decreased at six months, but move back to baseline values there were no differences by sex. Diastolic blood pressure decreased after six months with a more significant change in females **(B)**. Changes in ultrafiltration were different, in males increased and in females had small decrements **(C)**. Inferior Vena Cava varied in opposite way, increased in females and decreased in males **(D)**. Left ventricle mass index and NT-proBNP changes are shown in **(E,F)**, there were no significant differences by sex.

Figure 2 shows changes observed in cardiovascular parameters. Changes in systolic and diastolic blood pressure are in panels A and B. Diastolic blood pressure showed a sustained reduction in both sexes, but the change was more marked but not statistically significant in males. Systolic blood pressure showed trend back towards baseline values after six months without differences by sex. Ultrafiltration had different trends by sex, in males ultrafiltration increased and in females it had small decrements. The most important changes were seen in inferior vena cava diameter (panel D) with clear increments in females and reduction in males. Changes in Left ventricle mass index had the same pattern than systolic blood pressure, reduction at six months and trend back to baseline values after that. NT-proBNP levels had significant decrements along time without differences among sexes.

## Discussion

Data herein presented show measurable quantities of icodextrin metabolites in blood of diabetic patients treated with icodextrin PD solutions. Concentrations of icodextrin metabolites are higher in female than in male patients and were related with increments in the inferior vena cava diameter, a marker of blood volume. Changes in diameter of vena cava were in parallel with a trend back to baseline of systolic blood pressure after six months of treatment but were not followed by increments in LVMI or NT-proBNP values. These data suggest that icodextrin dose used may be excessive for women size, may be insufficiently metabolized by diabetic women, perhaps reflecting a smaller number of α-Amylase gene copies in this population (Viljakainen, et al., 2015; Nakajima, 2016), and/or decreased of α-Amylase activity (Al-Akl et al., 2021).

Icodextrin PD solutions have been successfully used since the 1990s. The colloid osmotic properties of icodextrin allows a sustained peritoneal ultrafiltration rate without the risk of excessive exposition to high glucose concentration in patients with fluid overload, a condition often seen in diabetic patients with high/fast peritoneal solute transport rate (Cho et al., 2013; Silver et al., 2014; Htay et al., 2018; Goossen et al., 2020).

In spite of its clinical value, some unexpected findings have been reported in patients treated with icodextrin solutions, among them increments in atrial natriuretic peptide, a cardiac hormone related with volume overload, and brain natriuretic peptide, another cardiac hormone related with volume and stretch overload as well as abnormal myocardial remodeling. Increments in LVM have also been reported. A plausible explanation for these findings is the accumulation of icodextrin metabolites in blood, which might exert a colloid osmotic effect and promotes expansion of blood volume (Bouchi et al., 2006; Davies et al., 2008).

Data from this secondary analysis are in line with the proposed explanation, icodextrin metabolites concentration increases along time. Small but significant increments were seen in all the measured size fragments. It is important to mention that only concentration and not the whole pool were measured since increments in blood volume was estimated indirectly. Early increments in icodextrin metabolites could be masked by increment in blood volume and be detectable until they accumulate along time and reach threshold of the technique used for the analysis. However, the decline observed in osmolality is an indirect support of the increment of icodextrin products, this mean displacement of other active molecules and ions accounting for osmolality (Mistry et al., 1994; Wolfson et al., 2002).

Explanations for the higher increments of icodextrin metabolites in women are not clear. Icodextrin pharmacokinetics follows a simple, single-compartment model that can be approximated by zero-order absorption and first-order elimination (Moberly et al., 2002), this means the main factors affecting its blood concentration are administered dose and hydrolysis or elimination. A standard volume of 2.0 L/Bag was used in all patients; this means the amount of icodextrin by body surface area was higher in females. On the other hand, net UF was lower among females; this picture might be secondary to increased intraperitoneal pressure which promotes more lymphatic absorption of peritoneal fluid and the icodextrin it contains. Intraperitoneal pressure was not measured; however, high fluid volume/body surface area ratio promotes more pressure and less UF (Paniagua, et al., 2004; Ventura, et al., 2000; Krediet, et al., 1988).

Another explanation derives from the efforts to find prognostic markers for the future development of obesity and diabetes. Biochemical and genetic research suggest that small number of genes copies encoding salivary and pancreatic α-Amylases confers higher susceptibility for this disease. Less number of α-Amylases genes copies is present in obese and diabetic patients compared with normal subjects. Furthermore, the number is even small in women than in men, which match with epidemiological studies showing higher prevalence of obesity and diabetes in women than in men (Nakajima, 2016; Viljakainen, et al., 2015). Differences in genetic pattern have not been found in some studies; however, lower serum α-Amylase activity is significantly associated with diabetes in women (Al-Akl, et al., 2021). Icodextrin comes into blood stream via lymphatic vessels, then it is metabolized by α-Amylase in small fragments, this means Icodextrin fragments depend on α-Amylase amount and activity (Olszowska, et al., 2019). Data from the present study are congruent with aforementioned findings. All patients were diabetic and differences in icodextrin metabolites higher in females, and α-Amylase activity was lower in females, the group which is expected to have a smaller number of α-Amylase gene copies.

And additional but not proved explanation is related enzyme activity. Macroamylase is an enzymatically active circulating complex of amylase bound noncovalently to immunoglobulins or to polysaccharides which is cleared from circulation by the kidney. Its size, interferes the bound of large substrates and consequently total activity decreases (Rosenblum et al., 1992). Contribution of Macroamylase to total α-Amylase activity in end stage renal disease patients is unknown. However, the presence of icodextrin could interfere its activity in hydrolyzing icodextrin itself and other small oligosaccharides. Icodextrin also exerts a competitive inhibition on normal α-Amylase. It is important to mention that in PD patients with regular use of icodextrin solutions, the plasma enzyme activity may decrease until 90% (Schoenicke et al., 2002). This means, the amount of available enzyme units is insufficient for icodextrin metabolism, which may be even more important in diabetic women who have low copies of α-Amylase genes and activity.

We do not know whether the observed concentrations of icodextrin metabolites completely explain increments in blood volume represented by increased inferior vena cava diameters. Calculations of the colloid osmotic pressure generated by icodextrin fragments indicates that 76% of pressure is generated by fragments larger than 8.5 kDa and small fragments of 2.1 kDa generates only 3% (Nishimura, et al., 2008). As was previously mentioned, blood volume was not measured and the pool of each fragment cannot be calculated.

Changes in blood pressure and Left ventricle mass index move according to concentrations of icodextrin metabolites; however, differences between sexes were not clear. Small sample size may explain the lack of statistical difference.

The study has some limitations including a small sample size. In spite of that, significant differences were seen in the most important findings. Other refers to indirect measurements of blood volume; inferior vena cava diameter has significant correlation with measurements with dilution methods and repeated measurements in the same patients reduce variability. Measurement of number of α-Amylase gene copies was not done; this evaluation requires complex technologies since the number of copies should be accompanied with evaluation of gene expression and the kinetic characteristics of the enzyme, particularly with its susceptibility of inhibition by icodextrin.

In summary, these data suggest that females are more susceptible to accumulation of icodextrin metabolites during long term PD treatment with standardized PD solution volumes, and that this might cause blood volume expansion, and return of blood pressure and Left ventricle mass index to baseline values. Further studies are necessary for the adequate evaluation of genetic and biochemical characteristics of α-Amylase as a possible determinant of icodextrin metabolites. Adjustments of icodextrin prescription on basis of diabetes, gender, and body size need also be analyzed.

## Data Availability

The original contributions presented in the study are included in the article/Supplementary materials, further inquiries can be directed to the corresponding authors.
